# Adaptation and feasibility assessment of a dating violence prevention program for girls in foster care

**DOI:** 10.1186/s41155-024-00292-4

**Published:** 2024-03-14

**Authors:** Julliane Quevedo de Moura, Manoela Mosena Saratt, Stephanie Caroline Souza da Silva, Victória Caroline Silva, Sheila Giardini Murta, Luísa Fernanda Habigzang

**Affiliations:** 1 Pontifícia Universidade Católica do Rio Grande do Sul, Prédio 11, 9º Floor, Room 924, Partenon, Porto Alegre, RS 90619900 Brazil; 2 Departamento de Psicologia Clínica, Universidade de Brasília, SQN 606, Campus Universitário Darcy Ribeiro, ICC Sul, IP-PCL Asa Norte, Brasília, DF 70910900 Brazil

**Keywords:** Prevention, Violence, Feasibility study, Adolescence

## Abstract

**Background:**

Dating violence in adolescence is a serious public health issue due to its significant impact on mental health and its significant predictive value for intimate partner violence in adulthood. Universal and selective programs can contribute to the prevention of this issue. Nonetheless, there are few selective programs with evidence of feasibility in contexts of social vulnerability.

**Objective:**

The present study examined evidence of the feasibility of a dating violence selective prevention program for girls in foster care by monitoring process indicators during the implementation phase of a pilot study.

**Methods:**

The program, originally designed for adolescents in the general population, was adapted to the context of girls at risk. The pilot study was conducted in the southern region of Brazil and involved the participation of six girls aged between 15 and 17. Both quantitative and qualitative measures were used, and the data were explored through frequency analysis, the Jacobson and Truax test, and content analysis.

**Results:**

The study identified favorable evidence regarding demand, acceptability, and adaptation of the intervention. On the other hand, contextual and institutional barriers hindered recruitment and restricted the reach of the intervention.

**Conclusion:**

Although there are changes to be made to improve the program’s applicability in its specific context, it should be emphasized that this study provides evidence to maintain the methods and content of the intervention.

**Supplementary Information:**

The online version contains supplementary material available at 10.1186/s41155-024-00292-4.

## Introduction

Dating violence is defined as a public health issue as well as an early form of rights violation and involves physical, sexual, economic, and emotional/psychological violence in intimate relationships among adolescents and young adults (Taquette & Monteiro, 2019; WHO, 2010). Additionally, it can be a precursor to violence in adult intimate relationships (Santos & Murta, 2019). Dating victimization is associated with various consequences, including low self-esteem and poor academic performance (Taquette & Monteiro, 2019), depressive and anxiety symptoms (Cava et al., 2020; Garthe et al., 2021), and risky sexual behavior (Kidman & Violari, 2018).

The literature shows concerning rates regarding dating violence victimization and perpetration in adolescence. An international survey conducted with 3711 participants, aged 12 to 18, reported that 1 in 3 adolescents experienced dating violence in the 12 months prior to the research. Among these findings, it is noteworthy that psychological violence was the most prevalent, followed by physical violence (Exner-Cortens et al., 2021). In the Brazilian context, Borges and Dell’Aglio (2020) study indicated that 93% of the 403 adolescents in the sample had already engaged in some type of violence in intimate relationships, with psychological violence being the most prevalent, followed by sexual and physical violence, respectively. Another study conducted with 305 Brazilian adolescents showed that 48% of the sample reported victimization, with psychological aggressions being the most prevalent form, followed by physical and verbal aggressions, respectively (Arnoud et al., 2023a, b).

This is a multi-cause phenomenon that can be better understood through the analysis of individual, relational, community, and social risk factors that interact and contribute to violence victimization and perpetration (WHO, 2010). According to the World Health Organization (2010), this phenomenon is best understood through the ecological model, which identifies age, gender, race, sexual orientation, and deficits in emotion regulation skills as some of the individual factors that should be considered for interventions. The relational risk factors indicated by the WHO (2010) include peer pressure, witnessing interparental violence, and jealousy. Although jealousy management has an individual component, it is understood in this model as a relational factor because it manifests itself in relation to other people. Regarding community risk factors, according to the ecological model, it considers limited or nonexistent violence response services, social vulnerability, and community poverty, among others. Finally, social risk factors such as gender inequality and cultural beliefs about violence acceptability and justification are emphasized (WHO, 2010).

Although the WHO has made progress by adopting the ecological model, it is observed that social determinants affecting health issues, such as violence, are still considered individual factors without the necessary critical and political analysis of systemic social inequities (Borde et al., 2015). Brazilian public policies focused on mental health and gender-based violence, aimed at girls and women, have not been clearly addressed as social determinants of mental health, thus indicating that there is an absence of intersectoral dialogue between these policies as a significant barrier to the prevention of violence and the development of proper mental health care (Medeiros & Zanello, 2018).

Gender, race, and class inequalities structure processes of social vulnerability and violence against specific groups. Social power asymmetries that are present in our society produce domination and subordination relationships that are at the core of violence and its consequences (Saffioti, 2004), including the restriction of access for girls and women to job opportunities, formal education, social mobility, and health services — phenomenon that can be understood as economic violence (Ohlan, 2021). Thus, dating violence is structured and sustained by macrosocial systems of oppression such as patriarchy, cisheteronormativity, racism, ableism, and ageism, which operate in interpersonal experiences of violence. In this sense, intersectionality theory contributes to a critical and in-depth analysis of violence dynamics, considering not only the existence of such systems of oppression but also the intersections that exist between them (Collins, 2022). Such intersectional analysis enables a critical understanding of specific processes of discrimination, violence, and barriers to accessing rights but also about resistance strategies, thereby highlighting and fostering emancipatory movements to overcome inequalities and pursue social justice (Bohrer, 2019).

Despite dating violence affecting boys and girls in the general population, empirical evidence indicates that social minority groups are more vulnerable to experiencing such violence. A study conducted by Courtain and Glowacz (2021) found that boys are more tolerant of violent attitudes within relationships than girls because they are encouraged culturally to resolve conflicts in a violent manner. In the American study (Roberts et al., 2018), conducted with 142 adolescents aged 13 to 19, girls from ethnic and racial minority groups were 2.7 times more likely to experience dating victimization. According to the Center for Disease Control and Prevention (2022), the risk of victimization is also higher for adolescents and young people in the lesbian, gay, bisexual, trans, queer, intersex, asexual, agender, pan/poli amorous, and nonbinary (LGBTQIAP+) community. This data is supported by Arnoud et al. (2023) study conducted with 350 Brazilian adolescents aged 16 to 19, which found that bisexual adolescents and non-cisgender adolescents had higher chances to report dating violence victimization.

Furthermore, contexts of social vulnerability, such as foster care, are also associated with a higher risk of experiencing dating violence. A study conducted with sheltered girls aged 13 to 18 emphasized that due to fragile family bonds, exposure to violence, and substance use, there was a greater risk of them being exposed to situations of vulnerability for sexual and reproductive health. The research also showed that these girls had a discourse of normalizing dating violence, in which gender norms, social exclusion, family, economic, and cultural issues were present in their realities (Penna et al., 2015). In Brazil, the study conducted by Arnoud et al. (2023a, b) identified that witnessing violence between parents or caregivers and lower income were predictors of dating violence victimization.

Also, in the Brazilian context, it should be noted that foster care is a reality for many families and occurs when there is a violation of rights, such as witnessing or directly experiencing domestic and family violence (Siqueira & Dell’Aglio, 2006). According to the Institute for Applied Economic Research (IPEA, 2021), 31,700 children and adolescents were removed from family life for protection in 2018, with two out of three of them living in the services offered in the Southeast and Southern regions. According to the report, there was an increase in girls among those in care, surpassing boys. Furthermore, regarding race, there is a predominance of black teenagers in foster care (IPEA, 2021).

The literature indicates that the reality of institutionalization has long-term effects, and when the family context has many adverse conditions, the care institution can become a protective context for this population (Robert et al., 2022; Silva et al., 2021; Siqueira & Dell’Aglio, 2006). Accordingly, making efforts to develop, adapt, and evaluate a dating violence prevention intervention for girls in foster care can provide new meanings in the realm of relationships and can offer tools for the interruption and prevention of violent episodes in dating relationships.

A literature review demonstrated that dating violence prevention programs are mostly universal, meaning they are aimed at the general population and are implemented in educational settings (Murta et al., 2013). Ten years later, this scenario has still not changed. A recent systematic review of the literature analyzed the production of dating violence prevention programs among adolescents, published in national and international journals from 2010 to 2021. It highlighted that out of the 54 articles found, 44 referred to universal programs, with only 3 from Brazil, and 10 programs were selective, all international (Moura, 2023).

In the Brazilian context, we are still in the early stages regarding the development and evaluation of these actions. Despite the guidelines of the American Psychological Association (2006) for the adoption of evidence-based interventions, gaps are observed between scientific production and practices implemented in public health policies (Menezes & Murta, 2018; Durgante & Dell’Aglio, 2018). This gap is even more evident when considering selective programs for the prevention of violence and health promotion for adolescents in socially vulnerable contexts.

A dating violence prevention program that has evidence of efficacy was developed by Murta et al. (2015) in Brazil. It is a multicomponent universal intervention applied in a school context with the main purpose of developing socio-emotional skills so that adolescents can identify violence in their intimate relationships and strengthen protective repertoires in this context. Adapting a universal prevention program for selective prevention is one way to initially fill this gap, with feasibility assessment being a recommended methodology for this process since simply transferring an intervention to a new context can be counterproductive, both ethically (e.g., it can cause harm or fail to benefit the target audience) and technically (e.g., it can represent a waste of resources) (Bowen et al., 2009; Durgante & Dell’Aglio, 2018).

Feasibility studies are recommended when there is no evidence that the intervention will benefit a specific group of people and when the intervention undergoes adaptations to meet the specificities of the target audience. It is a design that allows the understanding of the process and results of new and/or adapted interventions. Therefore, it is possible to explore the potential of the intervention, to provide evidence to understand if it is applicable or even necessary in a particular context, to verify if it meets the demands of the target population, and if it requires adjustments or reformulations in its structure and/or content (Durgante & Dell’Aglio, 2018). The analysis of the process becomes central through the following indicators: (1) demand, (2) acceptability, (3) implementation, (4) feasibility, (5) integration, (6) adaptation, and (7) limited-efficacy testing (Bowen et al., 2009).

It is worth noting that the process of adaptation for the selective context mainly requires an understanding of the specific requirements of this target audience, which has a history of rights violations and needs different management and dynamics than adolescents in the general population. Participatory/collaborative research insights are recommended for understanding the material reality in which the intervention is going to be applied and to allow theoretical development of interventions (Furtado et al., 2021). The involvement of researchers in actions and researched spaces, as well as the analysis of demands through focus groups, are recommended strategies. Therefore, this study aimed to adapt and examine the feasibility of an intervention for the prevention of dating violence for girls in foster care.

## Methods

### Participants

The participants in this study will be described in two stages: (1) intervention adaptation and (2) feasibility assessment. In both stages, convenience sampling was employed (Gray, 2012).

#### Stage 1: adaptation of the intervention

In the intervention adaptation study, two focus groups were conducted to analyze the needs of the target population. The first focus group consisted of adolescents from a foster care institution. Eleven girls were referred by the institution to participate; however, five dropped out after reading the consent form. Therefore, the group was formed by six participants. Inclusion criteria for the focus group were as follows: self-identifying as female, being between 14 and 18 years of age, being in a care situation, and voluntarily wanting to participate. The second focus group was composed of educators from the institution. Six educators were referred to participate; however, one participant requested to leave after the focus group had started. Inclusion criteria were as follows: being an educator at the care institution and voluntarily wanting to participate.

#### Stage 2: implementation and feasibility assessment of the intervention

The six girls from the care institution who participated in the focus group expressed interest in participating in the intervention. Therefore, all six were included in the pilot study. Pilot studies of interventions are indicated to examine the feasibility and the implementation, evaluating aspects such as the following: the attractiveness, clarity, and cultural relevance of content and procedures and the complexity, advantages, and disadvantages of materials (Bartholomew et al., 2011). The sample size should address these questions, without the requirement of sample representativeness for generalizing the findings to the population.

The girls were between the ages of 15 and 17. In terms of race, five girls self-identified as Black, and all the participants attended public schools. All the girls reported having had a previous intimate relationship. Four girls reported having relationships with both boys and girls. Of the six participants who started the program, only one completed it with 100% attendance. The respective participant was 15 years of age, had been in care for 6 years, self-identified as Black, was a 1^st^-year high school student in a public school, and had previous experiences of dating and being in relationships with both boys and girls. The reasons for the other participants’ withdrawal from the program were as follows: psychiatric hospitalization (1), a change in school schedule that made it impossible to attend the remaining sessions (1), adoption (1), returning to their original home (1), and refusing to continue participating because the topic caused discomfort (1).

### Instruments

#### Sociodemographic questionnaire

An 11-item questionnaire developed by the team with the aim of collecting information about the participants, such as gender, age, ethnicity, educational background, and sexual orientation.

#### Focus-group guide

The focus group was used because it is a methodology that allows to emphasize the understanding of the problems from the perspective of the target population groups, thereby increasing the chances of intervention success (Iervolino & Pelicioni, 2001).

- Focus-group guide for adolescents: Developed by the team to understand the requirements of the adolescents in relation to the topic of dating violence, as well as the program’s structure — for example, number of sessions, duration, schedule, and relevant topics. Thirteen guiding questions were used. Additionally, two vignettes containing stories of fictional characters in situations of dating violence were presented in order to obtain the perception of the adolescents about the theme, as shown in the following question: “What should be done to prevent violence in dating relationships?”
- Focus-group guide for the professionals/educators: Developed by the team to understand the requirements of the adolescents regarding the program through the perspective of the institution’s educators. Aspects related to the program structure, such as number of sessions, duration, schedule, relevant topics, and possible strengths and barriers to be encountered by the team, were addressed. Fifteen guiding questions and 1 vignette were used.

#### Conflict in adolescents dating relationships (CADRI)

This instrument consists of 70 items, of which 25 assess violence experienced, 25 assess violence perpetrated, and 20 items pertain to nonviolent conflict resolution strategies. The scale was adapted and validated for Brazilian Portuguese by Minayo et al. (2011). The instrument measures the violence present in romantic and sexual relationships among adolescents on a Likert scale from 0 to 3, where 0 = never, 1 = rarely, 2 = sometimes, and 3 = often. Cronbach’s alpha analysis revealed good internal consistency for most types of violence. The coefficient for the sub-scales of violence experienced was 0.87, and for the sub-scales of violence perpetrated, it was 0.88.

#### Attitudes about Aggression in Dating Situations Scale (AADS)

This 12-item scale aims to assess attitudes about dating aggression among adolescents. It was originally developed by Slep et al. (2001) and adapted for Brazil by Moura (2023). Its goal is to measure the justification of each type of aggression, and participants need to evaluate their degree of agreement with the aggressive behavior described in each situation on a 6-point Likert-type scale ranging from 1 (strongly agree) to 6 (strongly disagree). The sub-scales demonstrated adequate levels of internal consistency, with attitudes about female dating aggression of *α* = 0.75, attitudes about male dating aggression of *α* = 0.74, and attitudes about aggression against peers in dating situations of *α* = 0.56. Due to the Justification of Aggression against Peers dimension consisting of two items, internal consistency was calculated using the Spearman-Brown correlation, yielding a value of 0.72.

#### Justification of Verbal/Coercive Tactics Scale (JVCT)

This 24-item scale is composed of two parallel sets of 12 items each, addressing the justification of male and female tactics. It was originally developed by Slep et al. (2001) and adapted for Brazil by Moura (2023). Its goal is to assess the interviewee’s attitudes regarding the justification of three different types of emotionally aggressive behaviors (verbal aggression, control behaviors, and jealous behaviors) directed at boyfriends and girlfriends. Responses are measured using a 6-point Likert-type scale ranging from 1 (justified in many situations) to 6 (not justified in any situation). The sub-scales demonstrated adequate levels of internal consistency, including female verbal aggression of *α* = 0.66, female control strategies of *α* = 0.65, female jealousy of *α* = 0.75, male verbal aggression of *α* = 0.66, male control strategies of *α* = 0.70, and male jealousy of *α* = 0.74.

Rosenberg Self-Esteem Scale (RSE): This scale, adapted and validated for Brazilian Portuguese by Sbicigo et al. (2010), measures adolescents’ self-esteem. It consists of 10 items, with 6 related to a positive view of oneself (1, 4, 7, 8, 9, and 10) and 4 related to a self-deprecating view (2, 3, 5, and 6). Responses are evaluated using a 3-point Likert-type scale, with 1 representing “never”, 2 representing “sometimes”, and 3 representing “always”. Higher scores indicate higher levels of self-esteem. The sub-scales demonstrated adequate levels of internal consistency, with 0.70 for overall self-esteem, 0.77 for the negative self-esteem sub-scale, and 0.76 for the positive self-esteem sub-scale.

#### Field diary

Comprised of facilitators’ records containing impressions and information related to the process of integration into the partner institution, participant recruitment, and intervention implementation. At the end of each session, the following aspects were reported: topics covered in the session, girls who were present, emerging themes, assessment of participant engagement, perceived challenges in the session, and other impressions.

#### Record of received dose (Murta et al., 2009)

Instrument used in the original intervention study to examine participants’ interaction with the intervention and their acceptability and to gain insights into the intervention’s mechanisms of action (Moore et al., 2015). Considering behavior change as a process (Hashemzadeh et al., 2019), participants were encouraged to express cognitive, emotional, and behavioral manifestations of daily practice. The form was administered at the beginning of each session, asking, “What have you put into practice this week from the intervention? Practicing can be in small steps: thinking, desiring, or acting… Write here: ….”

#### Session satisfaction scale (Murta et al., 2016)

Like the previous instrument, this was used in the original intervention study to investigate how participants interacted with the intervention. Participants’ (dis)satisfaction responses regarding the intervention can inform about the program’s acceptability and its mechanisms of action (Moore et al., 2015). A form is used to evaluate the degree of participants’ satisfaction in the session. Originally containing eight faces expressing emotions designated by qualifiers such as “amazing”, “great”, “good”, “very good”, “OK”, “bad”, “horrible”, and “terrible”, it was adapted by the team to include five faces expressing emotions designated by qualifiers: “horrible”, “bad”, “OK”, “good”, and “great”.

#### Motivation ruler

Created by the team to gauge the level of motivation for the upcoming session. It features an image of a ruler specifying numbers from 0 to 10 to answer the following question: “How motivated do you feel for the next meeting?”, where 0 = not motivated at all and 10 = very motivated. Similarly, to the previous instruments, this instrument sought to describe participants’ response to the intervention and assess its acceptability through motivation for engagement or dropout.

### Data collection procedure

The study was conducted at a care institution located in the city of Porto Alegre, Rio Grande do Sul, Brazil, which serves children and adolescents who have been removed from their families by judicial order due to rights violations. Initially, contact was made with the institution to present the project. Subsequently, two visits to the location were conducted so the research team could familiarize with the institution’s structure, as well as meet the adolescents. Data collection lasted for a total of 5 months and took place in the year 2022. The procedures will be described in stages: (1) adaptation of the intervention and (2) implementation and feasibility assessment of the intervention.

#### Stage 1: adaptation of the intervention

A focus group was conducted with six adolescents from the institution to understand the requirements of the target population for the purpose of adapting the intervention. The group session was held in a single meeting, at 7:00 PM, and lasted for 1 h 30 min. Additionally, a second focus group was conducted with five educators from the institution to collect characteristics and requirements of the girls being attended. This group session was also held in a single meeting, at 5:00 PM, and lasted for 1 and a half hours. The meetings took place in rooms provided by the institution and were audio-recorded with the participants’ permission for subsequent transcription and analysis. Both groups took place in May 2022. Based on the qualitative analysis of the material, adaptations were made to the universally designed program to address the specific requirements of the target population in this study. The adaptation was based on the program originally developed by Murta et al. (2015).

#### Stage 2: implementation and feasibility assessment of the intervention

Following the completion of Stage 1, the intervention with nine sessions was implemented in a group format, with a weekly frequency and a duration of 1 h and 30 min. Table 1 shows the intervention description according to the Template for Intervention Description and Replication (TIDieR) checklist. The checklist is a guide developed by a group of experts to improve the completeness of reporting and replicability of interventions (Hoffmann et al., 2014).

**Table 1:** Intervention description and replication (TIDieR) checklist

**Item**	**Description**
**1. Brief name**	Dating violence prevention program for girls in foster care
**2. Why**	Intervention programs can help prevent violent episodes in intimate relationships among adolescents and, in the long term, prevent violence in intimate relationships in adulthood. There is a gap in Brazilian dating violence prevention programs targeting populations at higher risk of victimization, such as girls with a history of domestic and family violence in foster care.
**3. What (materials)**	The adaptation to a selective intervention was based on the multicomponent program originally developed by Murta et al. (2015), which is grounded in Bandura’s social cognitive theory (Bandura, 1977). The materials, such as games and prompts, used in this adapted program can be requested from the lead author.
**4. What (procedures)**	The detailed activities and procedures can be found in Additional file 1
**5. Who provided**	The focus groups and the nine intervention sessions were conducted by a psychologist specialized in cognitive-behavioral therapy and with a master’s degree in clinical psychology. Three psychology students acted as assistants. The students received prior training for their roles. The team received weekly supervision from two research professors who guided this study. One of them is the author of the original intervention.
**6. How**	The program was conducted in nine in-person group meetings.
**7. Where**	The space provided by the hosting institution was used, and it already included tables and chairs.
**8. When and how much**	The implementation of the selective program consisted in nine weekly sessions, each lasting 1 h and 30 min. The program happened in 2022.
**9. Tailoring**	Can be identified in Table 3
**10. Modifications**	The intervention was altered. Each session had 10 additional minutes, and a new theme, “testimony of violence in romantic relationships among family members and partners,” was included. Games and strategies to encourage discussions were adapted for the current context (e.g., the use of music and the creation of cards used to identify violent situations). All alterations can be found in Table 3.
**11. How well (planned)**	Adherence or fidelity to the original intervention was not assessed. However, supervisions, notes, and team trainings were conducted to promote fidelity of implementation.
**12: How well (actual)**	Adherence or fidelity to the original intervention was not assessed.

The sessions started at 7:00 PM on Thursdays in a group room provided by the institution. The intervention was conducted between the months of June and September 2022. The group was coordinated by a clinical psychologist and psychology students with prior training. The administration of the pre-test instruments was conducted during the first session of the program, while the post-test instruments were administered during the final session. These assessments took place in a group format with the assistance of the research team that conducted the intervention. Some process evaluation instruments were applied during the weekly sessions. Additionally, the facilitators made entries in a field diary. The application of the instruments can be seen in Table 2.

**Table 2:** Application of instruments

Instrument	Pre-test	After session	Post-test
Sociodemographic questionnaire	X		
Conflict in adolescents dating relationships (CADRI)	X		
Rosenberg Self-Esteem Scale (RSE)	X		X
Attitudes about Aggression in Dating Situations Scale (AADS)	X		X
Justification of Verbal/Coercive Tactics Scale (JVCT)	X		X
Record of dose received		X	
Session satisfaction scale		X	
Motivation ruler		X	
Field diary		X	

### Ethical procedures

The study obtained approval from the Research Ethics Committee of the Pontifícia Universidade Católica do Rio Grande do Sul, under authorization number 2.656. The project had the consent of the care institution. All participants were informed about the nature and purposes of the research and signed appropriate consent and assent forms tailored to their specific circumstances.

### Data analysis

The analysis of textual data related to the adaptation of the intervention was conducted through content analysis (Bardin, 1995). This analysis was conducted by two judges in three stages, which were as follows: (1) pre-analysis of the content of the focus group transcripts and field diary, (2) exploration and decoding of the material, and (3) data processing and interpretation. Bardin’s content analysis is one of the most commonly used methodological techniques in qualitative research and aims to find answers to a problem through scientific procedures (Bardin, 1995; Sousa & Santos, 2020). Data related to participant satisfaction and frequency were organized based on frequency counts. The feasibility analysis of this study was carried out according to the criteria proposed by Bowen et al. (2009): (1) demand: involves assessing the needs of the target population and evaluating whether the activities or components of the intervention meet these needs; (2) acceptability: evaluates whether the intervention is satisfactory and appropriate for those who received and applied it; (3) implementation: concerns evaluating whether the intervention can be fully implemented as planned within the context or if there are other factors that facilitate or hinder this; (4) feasibility: assesses the possibility of implementing the intervention with the necessary resources, means, circumstances, intensity, duration, and frequency; (5) integration: assesses whether implementing the intervention into existing infrastructure requires organizational changes; (6) adaptation: involves observing necessary modifications to the content, structure, implementation processes, and program evaluation, evaluating whether the intervention is compatible with the target audience; and (7) limited-efficacy testing: involves checking preliminary aspects of efficacy criteria, such as the effect on important variables.

The content analysis of the focus groups and the motivation scale were used to assess demand (1); the field diary, session satisfaction scale, dose received, and attendance frequency were used to assess acceptability (2); and the field diary was used to assess implementation (3), feasibility (4), integration (5), and adaptation (6). To assess (7) limited-efficacy testing, the Jacobson and Truax test, known as the JT method, was used. The JT method allows the effects of the intervention to be investigated by comparing the participant with themselves before and after the intervention in the evaluated dimensions. This way, it can classify whether the participant showed clinically relevant improvements, whether there was any harm for the intervention participants, or if no significant effects were observed (Del Prette & Del Prette, 2008; Jacobson & Truax, 1991). The benefits of the intervention were assessed by calculating the reliable change index (RCI) of the participant. The RCI helps indicate whether the changes between pre- and post-intervention are a result of the intervention. Changes are categorized as clinically relevant positive changes in situations where the difference between pre- and post-assessment is at least two standard deviations above the pre-intervention mean (Del Prette & Del Prette, 2008).

## Results

The results are described in two stages: (1) adaptation of the universal intervention to the selective prevention level and (2) evidence of the intervention’s feasibility.

### Step 1: adaptation of the intervention

The adaptation stage of the intervention comprised two axes: (a) results from the focus groups and (b) adaptation of the universal intervention to the selective prevention level.

#### Focus groups

Two focus groups were conducted, one exclusively for the girls and another for the educators. The focus group with the adolescents aimed to investigate the perspective that the adolescent girls had regarding dating violence and their specific requirements for the appropriate proposal of the intervention. Among the findings from the analysis, it was identified that the adolescents were interested in participating in the intervention on the topic of dating violence, as can be seen in the following statement: “For me, it was interesting to talk about this because not many come here looking for or wanting to know about this topic with us”. It was also possible to observe that they were familiar with expressions of violence in intimate relationships, as can be identified in the following statement: “My grandfather is jealous of my grandmother; he is explosive. He drinks and beats her, abuses her”. Additionally, the focus group highlighted the need for frequent sessions, equating to weekly sessions lasting for more than an hour, starting at 7:00 PM to accommodate the institution’s dinner schedule, with an average of 10 meetings. The sessions should cover topics such as issues related to sexual and gender diversity; racial issues; physical, verbal, and emotional violence; and a history of family and peer violence. It should be noted that many girls reported having witnessed violence in their homes, whether it was mistreatment or witnessing violence in the intimate relationships of parents or other family members.

The group aimed to understand the perceptions and previous knowledge that the educators had about dating violence and how this phenomenon was identified through the work they do with the girls. Furthermore, it sought to obtain their opinion regarding the structure of the program to be implemented in the institution. Among the findings, it was identified that the educators had knowledge about violent dynamics in adolescent dating, and they indicated the need for the program in the institutional context, as there were cases within the girls’ shelter who had experienced this violence. Furthermore, the analysis of the focus group revealed that in the educators’ perception, a weekly group would be exhausting, so they suggested a biweekly frequency, starting at 7:00 PM (to adhere to the house’s schedule), lasting approximately 50 min, covering topics related to sexual orientation, body expression, sexuality, strong libido (sic), self-esteem, self-awareness, aggressiveness, and behavior change. Additionally, the group highlighted the need to introduce different games and dynamics for the adolescents. Among the possible difficulties that the facilitators would encounter, the participants predominantly mentioned the potential resistance of the adolescents in addressing the topic. They also mentioned the need for persistence on the part of the program facilitators regarding the inherent difficulties of the context and the characteristics of the adolescents. Finally, among the suggestions, post-intervention feedback to the educators was predominant.

#### Adaptation from universal to selective prevention level

Following the placement in the facility and the conduct of the focus groups, analyses were performed, and team discussions were held to adapt the universal-level dating violence prevention program, aimed at the general population of adolescents, to the selective-level prevention, targeting adolescents in institutionalized contexts. The adaptation process from the universal intervention to the selective level was based on the program developed and evaluated by Murta et al. (2015). The original intervention consists of nine sessions, lasting 1 h and 20 min each, and was conducted in a school setting during the school day, targeting both boys and girls in the general adolescent population (Murta et al., 2015). The adapted intervention for the selective level retained the nine sessions, however, adjusted the duration to 1 h and 30 min, held in the evening, and conducted in a foster care context focusing exclusively on girls. Additionally, it adapted some sessions and dynamics to include themes identified as requirements in the focus groups. The adaptation of the intervention can be visualized in Table 3.

**Table 3:** Adaptation of the intervention from universal to selective prevention level

Sessions	Universal (objectives, duration, and techniques)	Selective (objectives, duration, and techniques)
1	Introduce the program to the participants, as well as the team of facilitators, and establish an initial rapport. Duration: 1 h 20 min. Activity: “similarities and differences”.	Present the program and the team, as well as establish an initial rapport. Introduce the adolescents, create the contract, and read the terms of consent. Conduct the pre-test instrument application. Duration: 1 h 30 min. Activity: group integration with the use of an interactive panel/poster.
2	Administer pre-test result assessment instruments and vote on preferred topics for upcoming thematic sessions. Duration: 1 h 20 min. Activity: presentation focused on constructing a bond.	Provide information about the emotional and cognitive aspects of passion. Facilitate the identification of responsive and abusive intimate relationships, whether from personal experiences or those of close people. Duration: 1 h 30 min. Activity: card game developed by the team focusing on abusive and violent situations
3	Promote the identification of responsive and abusive intimate relationships and provide information about the emotional and cognitive aspects of passion. Duration: 1 h 20 min. Activity: drawing session about being in love, passions worth pursuing, and passions not worth pursuing.	Promote reflection on the myth of romantic love and encourage recognition of jealousy in romantic relationships. Develop critical thinking about the costs and benefits of unhealthy coping mechanisms (violence, resignation) and raise awareness of healthy ways to cope with jealousy in dating (assertive communication). Duration: 1 h 30 min. Activity: use a song about jealousy as a trigger for discussion.
4	Promote the recognition of jealousy in dating, develop critical thinking about the costs and benefits of unhealthy coping mechanisms, and raise awareness of healthy ways to deal with jealousy (assertiveness). Duration: 1 h 20 min. Activity: reading a comic book story titled “Léo and Bia”.	Address the testimony of violence in romantic relationships among family members and partners, promote critical thinking about dysfunctional aspects of these relationships, highlighting legacies of relationships you wish to maintain and those you wish to change. Duration: 1 h 30 min. Activity: segment 1: balloon exercise: legacies I want to keep and legacies I want to change. Segment 2: For those in a relationship (satisfaction chart) and for those not in a relationship (exercise: how I would like my relationship to be) based on exercises from “Guia Diferenciando baladas de ciladas” (Murta et al., 2011).
5	Promote consequential thinking and self-awareness of emotions in decision-making situations and encourage self-care when facing decisions involving risks. Duration: 1 h 20 min. Activity: advantages and disadvantages of actions in dating using posters.	Promote critical thinking about gender roles and sexual and reproductive rights. Duration: 1 h 30 min. Activity: board game created by the team on “myths and truths”.
6	Develop initiatives for self-protection and problem-solving skills, foster empowerment (increasing personal resources), and recognize emotions and ways of dealing with emotion-focused problems. Develop critical thinking about problem-solving methods traditionally associated with femininity (passivity and submission) and masculinity (impulsivity and violence). Duration: 1 h 20 min. Activity: balloon game.	Address gender identity, sexual orientation, and race and their relationship with dating violence situations. Duration: 1 h 30 min. Activity: situational cards, vignettes as triggers for role-play/dramatization, and collective creation of a diversity mask.
7	Promote critical thinking about gender and sexual orientation roles and introduce sexual and reproductive rights. Duration: 1 h 20 min. Activity: myths and truths exercise based on “Guia Diferenciando baladas de ciladas” (Murta et al., 2011).	Develop initiatives for self-protection and problem-solving skills and favor the identification and increase of personal resources to address dating violence situations. Duration: 1 h 30 min. Activity: emotion and behavior traffic light, developed by the team.
8	Promote self-awareness of unpleasant emotions, raise awareness of how feelings are experienced, and provide information about assertive expression skills. Duration: 1 h 20 min. Activity: presentation of emotions.	Recognize the potential family, social, and legal support services in dating violence situations. Duration: 1 h 30 min. Activity: construction of a social support map.
9	Administer post-test assessment instruments, qualitatively evaluate participants’ progress throughout the program, and conclude with a celebratory tone. Duration: 1 h 20 min. Activity: “suitcase” dynamic about acquired strategies and a celebratory gathering.	Administer post-test result assessment instruments, qualitatively evaluate participants’ progress, and conclude the program with a celebratory tone with the adolescents. Duration: 1 h 30 min. Activity: group application of the instruments and celebration.

### Stage 2: evidence of intervention feasibility

The stage of evidence of feasibility for the pilot study will be described in two axes: (a) evidence of demand, acceptability, implementation, practicality, integration, and post-implementation adaptation (Table 4) and (b) initial evidence of pilot study efficacy (Table 5).

**Table 4:** Evidence of intervention feasibility

Evidence	
Demand	All participants in the focus group expressed a desire to participate in the program The mean motivation level of the participants in the group (*N* = 5) was 8
Acceptability	Participants showed a high degree of satisfaction with the sessions The girls were actively engaged in the activities Participants demonstrated improvements in coping strategies and the identification of dating violence, according to a qualitative assessment of the dose received
Implementation	Interference from other household activities coincided with the group’s schedule Participant attrition occurred due to situational reasons related to their homes, including psychiatric hospitalization, adoption, and returning home There were initial difficulties with room allocation and key management Session cancellations occurred due to a COVID-19 outbreak in the institution, with subsequent absences in the following week due to the virus Family phone calls coincided with the group’s schedule
Practicality	Basic infrastructure existed to enable implementation but could benefit from improvements such as better ventilation in the room The availability of audiovisual resources would enable other activities, such as the use of videos Participant attrition continued due to situational reasons related to their homes, including psychiatric hospitalization, adoption, and returning home
Integration	Recruiting participants from the institution was challenging due to the COVID-19 pandemic There was a perception of low institutional mobilization to inform the girls about the hours established for the group Lack of institutional involvement in inviting more girls for simultaneous group sessions Session cancellations were made on the last Thursday of the month due to the institution’s party for the birthdays of the month There was a need to adapt the group’s operating hours according to the household routine There was a need to address topics such as the transgenerational nature of family and peer violence Activities needed to be updated to include current elements like music and games that resonate with the adolescents’ reality
Adaptation

**Table 5:** Analysis of the JT method for the AADS and JVCT dimensions

Variable	T1	T2	RCI	
	*M*	*M*		
1. FAJ	3.50	4.25	0.90	NC
2. MAJ	5.00	6.00	1.00	NC
3. PAJ	5.50	6.00	0.48	NC
4. FVA	3.75	4.50	0.87	NC
5. FCS	4.50	5.00	0.53	NC
6. FJ	1.00	2.25	2.80	PCI
7. MVA	4.50	4.50	0.00	NC
8. MCS	4.50	5.00	0.53	NC
9. MJ	1.00	2.25	2.80	PCI

*M* Mean, *T1* Before the intervention, *T2* After the intervention, *RCI* Reliable change index, *PCI* Positive change index, *NC* No change, *FAJ* Female aggression justification, *MAJ* Male aggression justification, *PAJ* Peer aggression justification, *FVA* Female verbal aggression, *FCS* Female control strategies, *FJ* Female jealousy, *MVA* Male verbal aggression, *MCS* Male control strategies, *MJ* Male jealousy

#### Evidence of intervention feasibility

As a result of these pieces of evidence, some recommendations for future interventions aimed at this population should be considered regarding the recruitment and retention of participants in the program. To achieve this, the following actions are suggested: the development of a strategy plan in collaboration with the institution to enhance participant retention, raising awareness within the institution about recruiting girls and the importance of simultaneous groups taking place, integrating and involving the research team in the recruitment and explanation of the program to the participants, including the research team in the process of inviting the participant to join the group, creating individual physical invitations to make it more appealing to the girls, and initiating the program with a larger number of girls due to the characteristic attrition of the context. It is noted that according to the evidence from this pilot, there is no need to alter the content of the sessions; however, reducing the number of sessions or conducting two sessions per week may be a strategy to address the contextual attrition of the shelter, which involves high turnover of adolescents undergoing situations such as adoption and returning home. Finally, the need for audiovisual resources in the group room and checking the institution’s calendar in advance for events occurring at the same time as the weekly sessions are also emphasized.

#### Initial evidence of pilot study efficacy

The preliminary efficacy evidence was assessed through a comparison of pre- and post-test instrument scores from the sole participant who completed the intervention. The participant, aged 15, self-identifies as a Black woman and maintains affective relationships with boys and girls. Additionally, she is enrolled in a public school and has been in foster care for 6 years. It was observed that the participant showed improvements in all evaluated dimensions. Therefore, the results demonstrated an increase in scores in the dimensions of justification of female aggression, male aggression, and peer aggression; dimensions of female verbal aggression, female control strategies, female jealousy, male verbal aggression, male control strategies, and male jealousy; and an increase in self-esteem levels (Table 5). Although an increase in all scores was observed, the results of the JT method indicated that reliable clinical change was observed in the dimensions of female and male jealousy (Table 5).

## Discussion

This study conducted the adaptation and feasibility evaluation of a dating violence prevention program for girls in foster care through a pilot study. Among the findings related to the demand, all adolescents showed interest in discussing the proposed topic. They also indicated the need to address experiences of violence witnessed in their families and discuss aspects related to sexual and gender diversity. Therefore, the focus group as a resource for identifying and analyzing requirements was crucial. This resource was also useful in a study on dating violence prevention for pregnant teenagers (Herrman & Waterhouse, 2014). According to Corrêa et al. (2021), the focus group is a methodology that allows for an emphasis on understanding problems from the perspective of the target population, increasing the chances of intervention success.

Similarly, as in the universal intervention (Murta et al., 2015), in terms of acceptability, the program showed good indicators. This result is consistent with the literature on dating violence prevention programs, which generally highlights the receptivity and acceptability of interventions on the subject for adolescent audiences (Rothman et al., 2020; Santos & Murta, 2019). In this study, acceptability was evidenced not only by the effective use of the sessions, as assessed through field diaries and observations, but also through the evaluation of the satisfaction of the participants with the sessions delivered. Out of the eight evaluations conducted, seven were “very satisfied”, and one was “satisfied”. The acceptability of an intervention by the target audience is one of the important indicators of its quality (Donabedian, 2003).

In the context of dating violence prevention interventions, it is highlighted that the multicomponent format of interventions is more effective than purely informational or psychoeducational interventions (Murta et al., 2013). In this context, interventions should provide resources that not only offer information passively but also incorporate playful and participatory strategies. This approach can help raise awareness about the non-normalization of violence and empower participants to learn how to manage conflicts within their own relationships (Priolo-Filho et al., 2021). The multicomponent use of different educational resources in this intervention, such as psychoeducation, individual activities, group dynamics, music debates, and homework assignments, may have contributed to the participants’ positive acceptance of the activities. According to Gottfredson et al. (2015), using different learning methods to benefit individuals is necessary to achieve better results in interventions, taking into account individual and cultural differences in acquiring new knowledge. In other words, the assessment of needs, as conducted in this study, is essential to understand the target audience, preventing the mere importation of disconnected activities that do not align with their concrete reality.

Discussions on dating violence considering the specificities of the phenomenon for LGBTQIAP+ adolescents were very well received by the group. Interventions focusing on violence in intimate relationships should not adopt a cisheteronormative and exclusionary perspective. LGBTQIAP+ adolescents are at higher risk of violence in romantic relationships and experience additional stressors such as stigma and discrimination that can aggravate bad mental health outcomes (Arnoud et al., 2023a, b; Whitfield et al., 2021). Nonheterosexual and non-cisgender adolescents suffer specific forms of violence in their relationships, such as invalidation of their gender identity and sexual orientation, threats from their partner to disclose their orientation, partners that may force them to “come out”, objectification and hypersexualization, corrective rapes, among others (Corey et al., 2023; Harden et al., 2022). In this regard, LGBTQIA+ adolescents encounter a phenomenon described in the literature as the “double closet”, meaning that there are additional barriers to revealing the violence experience because that can also mean revealing one’s own sexual orientation (Vickers, 1996). Thus, programs with cisheteronormative content imply lower adherence of the participants and may represent a new form of aggression for LGBTQIAP+ adolescents (Broadway-Horner & Kar, 2022). Taking into consideration diversity in violence prevention programs allows participants to feel represented throughout the intervention. In this specific study, the majority of participants shared having dating experiences with boys and girls and have expressed satisfaction with the proposed activities that took into consideration different configurations of relationships and not only cisheterosexual dynamics. Thus, dating violence prevention should also promote health and act in a way that the rights of the groups that are historically marginalized in both, at the community level and in psychological studies, can be ensured.

Despite the acceptability and receptivity of the intervention’s theme by the adolescents, as evidenced in the focus group and session evaluations, the topic of the fourth session, family violence, led to a participant dropping out. The participant mentioned that the topic made her uncomfortable because she had experienced many such situations in her life. According to Rojas et al. (2020), a traumatic event experienced or witnessed, especially in childhood and adolescence, can have emotional and behavioral consequences for development. These consequences include the avoidance of situations that evoke traumatic memories. Accordingly, it is emphasized that interventions addressing topics related to violence and sensitive subjects in general can generate emotional discomfort, leading to nonacceptance by some participants. Adolescents may not feel safe and/or comfortable sharing adverse experiences in the group, and this decision should be respected. It is also essential for intervention facilitators to seek to investigate whether participants in these conditions have mental health support; otherwise, necessary referrals should be made.

Concerning the implementation, it was expected that some barriers would be encountered due to the context of the COVID-19 pandemic. As expected, the pandemic scenario in this case delayed the start of group activities by 2 years, impacting the project’s planning and execution. The pandemic also affected the program after it started, as one session had to be rescheduled due to a virus outbreak at the institution, leading to a lack of participants in the following session. COVID-19 has deepened social inequalities in Brazil, and there were no efforts by the government to promote policies that could prevent violence and promote health of adolescents in contexts of social vulnerability, such as those in foster care. In fact, data shows that the dismantling of existing social policies and cuts of funds that should be allocated to the social assistance Brazilian system had an immense impact on foster care institutions (Boschetti & Behring, 2021). The pandemic has changed the landscape of cities, routines, education, and academic research (Oliveira, 2021). Among these impacts on the academic lives of researchers was the shift from real-life to online activities. Interventions for the prevention of dating violence online have been identified in the literature, such as the study by Rothman et al. (2021) focused on the autistic adolescent population. However, changing this intervention to an online format would not have been viable due to the institution’s lack of infrastructure and would have made many of the planned techniques unfeasible.

Regarding the practicality indicator, the results highlighted some compromises. This indicator aims to assess the possibility of implementing the intervention with adequate material and human resources, means, circumstances, intensity, duration, and frequency. Although the intervention had low costs in terms of materials used and managed to deliver the nine proposed sessions, the results indicated the need to construct conditions that increase institutional support. Through field diaries and participant attendance analysis, it was noticed that attrition was influenced by specific local circumstances at the institution. In these situations, inadequate working conditions posed obstacles to the institution’s capacity to include girls in the group at the appropriate time. These challenges included difficulty accessing the group room, insufficient communication from the institution regarding concurrent activities, and a lack of information regarding the adoption process for participants, the return to their original homes, and psychiatric hospitalization.

Regarding these external circumstances that affected the foster care scenario and led to attrition, it can be highlighted that, in addition to aspects involving institutional support, aspects related to the mental health of these young women also require attention. During the program, two participants experienced impairments as a result of side effects from psychiatric medication. Subsequently, one of these participants withdrew from the program due to psychiatric hospitalization, which was not her first such hospitalization. It should be noted that she was very participative in the sessions from the beginning and showed interest in the theme; however, this circumstance led to her withdrawal. According to Winkelmann et al. (2021), during the period of foster care, young people express the consequences of the rights violations they have experienced in various ways, with medicalization and psychiatric practices often constituting the responses to this reality. The Brazilian context shows that adolescents under the jurisdiction of the justice system are more likely to be subjected to psychiatric hospitalization than adolescents who are not in foster care. Despite reforms in mental health care policies in Brazil, a hygienist approach persists toward socially marginalized groups. Therefore, it is emphasized that there are challenges in the adolescent protection system, especially for those with mental health needs in foster care (Oliveira et al., 2018).

Regarding mental health and provided assistance, it is important to emphasize that the majority of children and adolescents currently in foster care in Brazil are Black, from low socioeconomic backgrounds, and that had experiences of domestic and family violence. Analyzing the recurring medicalization and psychiatric hospitalization for this specific group requires considering the intersection between racism and sexism that affects black Brazilian girls, generating negative effects on mental health. Systematic reviews on racism and mental health denounce institutional racism as a historical form of oppression and social control through the medicalization and asylum confinement of Black and poor women. Additionally, the reviews point to the lack of analysis of the effects of racism on health care provided by public services. A study conducted by Cândido et al. (2022) analyzed 220 medical records of self-identified Black children and adolescents treated at Psychosocial Health Care Centers in Brazil and found that 27% of the cases had a history of violence (Cândido et al., 2022). Therefore, strategies for promoting health and strengthening community ties should be prioritized in mental health policies for this group, considering intersections between racism and sexism and its relationships with experiences of violence and impacts on mental health.

The integration indicator presented some impairments, including session cancellations due to both the COVID-19 pandemic and the birthday celebration of the institution, which resulted in weeks of unexpected implementation delay. Requests to hold the intervention on an alternative day to this fixed monthly event were denied. Aspects related to the timing of the group, which was in the evening, may have also negatively impacted the program’s integration into the institution. Furthermore, the program’s integration into the infrastructure could be improved, such as obtaining audiovisual resources in the group room and a location with better ventilation.

Similarly, as in the universal intervention that was conducted in a school (Murta et al., 2015), this intervention presented difficulties related to the institutional context. Both the practicality and integration indicators were affected by institutional issues in the care institution. According to Piske et al. (2018), the time and space of institutional coexistence are not always easy spaces for research, and environmental and social problems in care institutions are also complex, as they relate to human relationships and working conditions. It is important for care institutions to develop actions to promote the health of children and adolescents under their care; however, conditions do not always make this feasible (Julião, 2020). The outsourcing and precariousness of work in the public services of social assistance are a reality that alters fundamental principles of the policy, generating negative effects for workers and users (Pereira et al., 2017). Deficient infrastructure, low resources for continuous training, and for the maintenance of the already existing services provided negatively affect the psychosocial development of foster care children and adolescents in Brazil.

Preliminary evidence of effectiveness measured by the JT test compared pre-test and post-test scores of the only participant who completed all sessions of the intervention. The findings showed that although an increase in all scores was observed, reliable clinical change was only observed in the dimensions of female and male jealousy. It should be highlighted that the original intervention, which this study sought to adapt, did not evaluate limited-efficacy testing. However, jealousy was the focus of one of its sessions (Murta et al., 2015). Given that reliable clinical change was observed in the female and male jealousy dimensions, it should be emphasized that jealousy is often understood as an expression of love, care, and attention among individuals and is naturalized in Western culture, therefore influencing young people’s romantic learning (Bryant, 2017). Future pre-test and post-test evaluations are recommended with participant groups to provide a more representative picture of the behavior of these variables.

In the Brazilian study by Ferriani et al. (2019), findings indicated that adolescents understand jealousy as an inherent phenomenon in relationships, being both a trigger for violence and a topic that can lead to discussions, aggression, and even death in the context of romantic relationships. In this intervention, jealousy was specifically addressed in session 3; however, the topic came up in other sessions, especially when brought up by one of the participants. Furthermore, jealousy was an aspect identified in the evaluation of the adolescents’ requirements, as many of them reported adverse situations related to jealousy. Therefore, jealousy is a component of the violent dynamics of dating relationships and should be considered in structuring prevention programs for this population (WHO, 2010).

Through the execution of a pilot study and its evaluation, aspects and barriers to the feasibility of a particular program can be identified, allowing the construction of solutions for the future implementation of preventive programs. Possible solutions to the problems raised in this study include changes in the intervention itself and in the institutional context. Regarding the intervention itself, to reduce participant attrition due to institutional factors, the program could start with a larger number of participants assigned to a group. Additionally, the program could be made shorter, as long as its objectives and content are preserved, or maintain the nine sessions but conduct them twice a week.

Considering the institutional context, there is a need to strengthen institutional relationships by raising staff awareness about the program’s importance and the girls’ participation. It is also recommended to encourage active participation from the staff in inviting the girls to take part in the sessions and to collaborate closely with educators toward this objective. Furthermore, creating invitation cards for the adolescents could be a way to attract them to the meetings and clearly establish the aim of the groups, not solely relying on the institution to handle this task. The program’s timing, which was in the evening, may have negatively impacted recruitment and the involvement of more girls; therefore, it is suggested that the program could be held at different times. Although there are changes to be made for better program applicability, it should be noted that this study provides evidence to maintain the methods and content of the intervention conducted.

## Conclusions

According to the findings, the program proved to be feasible in terms of acceptability, demand, and adaptation. However, “It is important to emphasize that this study has some significant limitations”. The COVID-19 pandemic had an adverse impact on the implementation of this intervention, reducing the time available for the research team to be in the institution, which deviated from the initial plan. Furthermore, the evidence in this study comes from a single group. It should be emphasized that the group conducted is part of a specific institution and may not be representative of other foster care institutions. Furthermore, it is noteworthy that the involvement of social educators in the recruitment of participants is a factor that may have affected greater adherence to the intervention by the adolescents. It is suggested that future studies organize participant recruitment through direct invitations from the research team to the adolescents.

The presented intervention demonstrates potential for replication taking into consideration its thematic and methodological structure. Intersections between race, gender, class, and sexual orientation must be considered in order to build an accurate understanding of the phenomenon of dating violence so that adolescents feel represented in their experiences of oppression and violence and can also develop strategies of resistance through emancipatory processes. It is suggested that future studies deepen evidence that can enhance aspects of implementation, practicality, and integration, as well as to obtain initial evidence of effectiveness with greater statistical robustness, by conducting group comparisons, for instance.

### Supplementary Information

**Supplementary Material 1.**

## Data Availability

We declare that the manuscript in question is original and has not been submitted to another journal. This is an unpublished work, having not been published in any other publication in full or in part, not even a work with substantially similar content, written by me. All data in the article is legitimate and authentic. The author confirms that the main data generated or analyzed during this study are included in this published article. Additional pertinent information supporting the results of this study is available upon request.

## Abbreviations

AADS: Attitudes about Aggression in Dating Situations Scale
CADRI: Conflict in adolescents dating relationships
COVID-19: Coronavirus disease
FAJ: Female aggression justification
FCS: Female control strategies
FJ: Female jealousy
FVA: Female verbal aggression
JVCT: Justification of Verbal/Coercive Tactics Scale
LGBTQIAP+: Lesbian, gay, bisexual, transgender, queer, intersex, asexual/agender, pan/poly, non-binary, and more
M: Mean
MAJ: Male aggression justification
MCS: Male control strategies
MJ: Male jealousy
MVA: Male verbal aggression
NC: No change
PAJ: Peer aggression justification
PCI: Positive change index
RCI: Reliable change index
RSE: Rosenberg Self-Esteem Scale
T1: Before the intervention
T2: After the intervention
WHO: World Health Organization
