# Supplementary material for: Adaptation and feasibility assessment of a dating violence prevention program for girls in foster care
Source: Psicol Reflex Crit. 2024 Mar 14;37:9. doi: 10.1186/s41155-024-00292-4 (PMC10940555; doi:10.1186/s41155-024-00292-4)
Supplement: Supplementary file 1 — Supplementary Material 1. [file 41155_2024_292_MOESM1_ESM.docx]

**Additional file 1**

**Session 1**

**Title:** What group are we?

**Theme:** Group integration

**Recommended time:** 1h30min.

**Purpose:** Present the program and the intervention team, as well as establish an initial rapport. Conduct a brief presentation of the participants, establish a collective agreement of rules for the proper functioning of the intervention, read the informed assent forms and administer the pre-test instruments. After the completion of these stages, a group dynamic titled "Who are we as a group" is performed in order to increase group integration.

**Techniques:** Use of panel/poster with active group construction

**Session description:** Initially, the program and team were introduced. Subsequently, the informed assent forms were presented and those adolescents who agreed in participating signed the document. Following this, the adolescents were prompted to introduce themselves. Then, the group contract with rules for the proper functioning of the intervention was collaboratively constructed with active participation from the adolescents, utilizing cardboard for recording contractual agreements. Next, the adolescents were divided into three subgroups for the collective administration of pre-test instruments, including a sociodemographic questionnaire, CADRI, EJCTC, AASN, and EAR. Upon completion of the pre-test instruments, the adolescents engaged in an integration dynamic. The dynamic consisted in creating a poster that could reflect the identity of the emerging group. Each adolescent inscribed their name alongside a word or image provided in print, symbolizing their response to the query: "Who am I and what group are we?" Subsequently, each participant elucidated the rationale behind their chosen representation. Finally, the adolescents were asked to provide a written evaluation utilizing the motivation ruler instrument, assessing their level of motivation for the forthcoming meeting by responding to the query: "How motivated do you feel for the next encounter?"

**Materials Required:** Cardboard, marker pen, colored pens, glue, assorted printed images of animals, objects, etc., copies of the motivation ruler instrument.

**Instruments/ Evaluation:** Sociodemographic questionnaire, CADRI, EJCTC, AASN, EAR and motivation ruler instrument.

**Session 2**

**Title:** Passions that are (not) worth it.

**Theme:** Identification of abusive and violent behaviors within dating context

**Recommended time:** 1h30min.

**Purpose:** To inform about the affective and cognitive aspects of passion. To promote the identification of responsive and abusive intimate relationships, whether in personal experience or in the experiences of individuals that are close to the participants.

**Techniques:** Card game about abusive and violent situations.

**Session description:** The adolescents were invited to participate in a card game. The game involved identifying each violent situation represented on the cards and placing/gluing them onto a poster containing typologies of violence: physical, psychological, sexual, and financial. At the end, the facilitators provided psychoeducation on how it feels being in love, what is dating violence and what relationships are worth pursuing. Finally, the adolescents were asked to complete a written evaluation by answering the following question: "What was your level of satisfaction with today's meeting?"

**Materials Required:** Cards, poster, marker pens, adhesive tape.

**Instruments/ Evaluation:** Written question to assess the level of satisfaction with the session.

**Session 3**

**Title:** Jealous, me??? I just take care of what's mine...

**Theme:** Myth of romantic love and jealousy.

**Recommended time:**  1h30min.

**Purpose:** Promote reflections on the myth of romantic love and recognize the experience of jealousy in dating, develop critical thinking about the costs and gains of unhealthy coping mechanisms (violence, resignation), and raise awareness for healthy coping with jealousy in dating (assertive dialogue).

**Techniques:** Use of a Brazilian popular song called "Ciúmes" by Jorge & Mateus" and another song called "Jireh - Ciúmes" by @Dan Lellis"

**Session description:** Two music options, from different genres, were suggested by the facilitators to the participants. A vote to choose the musical genre was conducted and the hip-hop/trap song " "Jireh - Ciúmes" by @Dan Lellis won. After listening to the song, the adolescents were given the printed lyrics and were invited to circle on the sheet with the lyrics the healthy and unhealthy attitudes of the relationship addressed in the song. Next, the relationships between the myth of romantic love and jealousy were discussed, as well as the identification of strategies to deal with them, and the advantages and disadvantages of these coping mechanisms. In this session, facilitators also conducted psychoeducation on assertive communication.

**Materials Required:** Electronic device for playing music, sheets with printed song lyrics, pens.

**Instruments/ Evaluation:** Written question to assess the level of satisfaction with the session.

**Session 4**

**Title:** Inheritance I want to maintain / Inheritance I want to change

**Theme:** Transgenerationality of family and peer violence.

**Recommended time:** 1h30min.

**Purpose:** Address witnessing violence in romantic relationships among family members and peers, promote critical thinking about dysfunctional aspects of these relationships and point out the inheritances one wishes to keep and those one wishes to change.

**Techniques:** Moment 1: Balloon dynamics: inheritances I want to keep and inheritances I want to change; Moment 2: for those in a relationship (satisfaction chart); for those not in a relationship (exercise on how I would like my relationship to be). Moment 2 is based on the exercises from the Guide "Distinguishing Nightclubs from Traps" (Murta et al., 2011).

**Session description:** Initially, the balloon dynamics were carried out. The participant received two balloons, one representing the inheritance she wanted to keep, and the other representing the inheritance she wanted to change. The participant received two papers to write down what those inheritances were and to later stick them onto the respective balloons. After this moment, the teenager was invited to talk about her inheritances, with the final goal being to keep the balloon with the inheritances she wanted and to pop the balloon with the inheritances she did not want to keep. The facilitators provided psychoeducation on the topic and closed the first dynamic. Afterwards, the last dynamic consisted of an exercise focused on current and/or future relationships. Adolescents who were intimately involved with someone were invited to evaluate their relationships, while those who were not in relationships sought to assess the characteristics they would like in a dating relationship, both based on the instrument from Chapter 6 of the guide "*Diferenciando Baladas de Ciladas*" (Murta et al., 2011). As the only participant who completed the session was not in a relationship at that time, she evaluated characteristics she would like to have in a relationship.

**Materials Required:** Balloons, pens, paper, adhesive tape, copies of the exercises from Chapter 6 of the manual "*Diferenciando Baladas de Ciladas*".

**Instruments/ Evaluation:** Instrument by Murta (2009) that evaluates received dose.

**Session 5**

**Title:** Let's talk about gender and sexual and reproductive rights?

**Theme:** Gender and sexual and reproductive rights.

**Recommended time:** 1h30min.

**Purpose:** Develop critical thinking about gender roles and sexual and reproductive rights.

**Techniques:** Board game about myths and truths regarding gender, coping strategies focused on sexual and reproductive rights, and identification of healthy or unhealthy aspects of relationships.

**Session Description:** The session started with the board game. The game had a starting line and the order of the adolescents was selected randomly and, according to that order, the girls rolled the dice and moved spaces. Each space had a question and the following answers: MYTH or TRUTH (based on Chapter 2 of the manual "*Diferenciando Baladas de Ciladas*"). Those who answered correctly moved forward. The first to reach the finish line is the winner. All the girls received a chocolate for participating in the competition. At the end of the game, the facilitators provided a reflection on the topics presented in the game.

**Materials Required:** Board game constructed by the research team, dice and chocolates.

**Instruments/ Evaluation:** Instrument by Murta (2009) that evaluates received dose.

**Session 6**

**Title:** Understanding other ways of loving

**Theme:** Diversity and violence (LGBTQIA+ community and race/ethnicity)

**Recommended time:** 1h30min.

**Purpose:** To address gender identity, sexual orientation, race and their relationship with violent situations within intimate relationships.

**Techniques:** Situational cards and vignettes that were used as triggers for role-play/dramatization; and a collective confection of a "diversity mask".

**Session description:** The session began with three cards related to abusive and violent situations in the context of diversity (gender/sexual orientation/race) used to trigger collective discussion. The participants were invited to dramatize these situations, focusing on contrasting performances: how to deal with these situations assertively and non-assertively. Finally, the facilitators distributed a blank cut-out template of a mask representing a face, and the participants were invited to assemble their masks using artistic materials provided by the team, aiming to represent the topics discussed.

**Materials Required:** Cards, mask, magazine, paints, brushes and yarn.

**Instruments/ Evaluation:** Written question to assess the level of satisfaction with the session.

**Session 7**

**Title:** Emotions and behaviors in dating traffic light

**Theme:** Crisis resolution strategies.

**Recommended time:**1h30min.

**Purpose:** To develop initiatives for self-protection and problem-solving skills, promoting the identification and increase of personal resources to act in the face of violent situations in dating.

**Techniques:** Emotions and behaviors traffic light: use of the poster made by the facilitating team that shows an image of a traffic light, with green, yellow, and red lights, signallingincreasing levels of alert for harmful situations in the relationship (continue, caution, and care, respectively).

**Session description:** The session begins with the traffic light dynamics in order to ease the collective discussion. Beside each color of the traffic light, the participants placed papers with situations experienced in dating relationships according to their severity. Later, the participants accessed a box containing shuffled slips of paper containing conflict resolution strategies. The teenagers picked a slip of paper from the box and placed it under the respective color of the light and explained why she chose that conflict resolution strategy. Finally, her strategies for dealing with the problems were discussed with the facilitators. The teenagers received sheets from Chapter 9: Seeking Solutions to Problems from the manual "*Diferenciando Baladas de Ciladas*" to take home as additional reading (Murta et al., 2011).

**Materials Required:** Poster of the emotions and behaviors traffic light and sheets with a copy of the chapter for additional reading.

**Instruments/ Evaluation:** Instrument by Murta (2009) that evaluates received dose.

**Session 8**

**Title:** Emergency button: who could help me?

**Theme:** Social support network.

**Recommended time:** 1h30min.

**Purpose:** To allow the recognition of potential social support network composed of family, social, and legal support in situations of dating violence.

**Techniques:** To build a social support network map.

**Session description:** The participant created a map with elements of family, social, health, and legal support networks. Images of various facilities (hospitals, schools, police stations, mental health centers, health centers, social assistance centers, etc.) were provided to be affixed to each map. The facilitators, together with the participant, engage in dialogue about the created map.

**Materials Required:** Copies of the exercise, images of public services facilities, glue, scissors, pencils, and pens.

**Instruments/ Evaluation:** Written question to assess the level of satisfaction with the session.

**Session 9**

**Title:** What do we bring from here?

**Theme:** Closure.

**Recommended time:** 1h30min.

**Purpose:** To administer post-test evaluation instruments, qualitatively assess the progress of the participants throughout the program, and conclude the intervention with a celebratory tone with the adolescents.

**Techniques:** Group administration of the post-test instruments and celebration with music, sweets, and snacks.

**Session description:** Initially, the program closure was explained, and subsequently, a rapport regarding the administration of the post-test instruments was established. The applied instruments were the following: EJCTC, AASN, and EAR. After this moment, a summary of the program was conducted, covering previous topics, and providing space for the adolescents to share their experiences and feelings regarding the program. Finally, a music player was made available, along with a table of sweets and snacks for the celebration.

**Materials Required:** Printed version of the instruments, pens, music player device, snacks and beverages.

**Instruments/ Evaluation:** EJCTC, AASN, EAR and Instrument by Murta (2009) that evaluates received dose.
